# Phelan–McDermid Syndrome in Pediatric Patients With Novel Mutations: Genetic and Phenotypic Analyses

**DOI:** 10.3389/fped.2022.888001

**Published:** 2022-08-23

**Authors:** Liang Chen, Zhi-ye Yao, Xiangtao Wu, Shao-ru He, Yu-mei Liu, Xue-yan Wang, De-zhi Cao, Xing-kun Yang, Jian-bo Zhao, Zi Ren, Hong Li, Zheng Pei, Hong-ke Ding, Zhi-chun Feng

**Affiliations:** ^1^Department of Neonatology, Guangdong Provincial People's Hospital, Guangdong Academy of Medical Sciences, Guangzhou, China; ^2^Department of Pediatrics, The First Affiliated Hospital of Xinxiang Medical University, Xinxiang, China; ^3^Prenatal Diagnosis Center, Chongqing Maternal and Child Health Hospital, Chongqing, China; ^4^Department of Neurology, Shenzhen Children's Hospital, Shenzhen, China; ^5^Prenatal Diagnosis Center, Foshan Maternal and Child Health Care Hospital, Foshan, China; ^6^Department of Neurology, Beijing Children's Hospital, National Center for Children's Health, Capital Medical University, Beijing, China; ^7^Center for Reproductive Medicine, Sixth Affiliated Hospital of Sun Yat-sen University, Guangzhou, China; ^8^Pediatric Center, Zhujiang Hospital of the Southern Medical University, Guangzhou, China; ^9^Department of Rehabilitation, Guangdong Women and Children Hospital, Guangzhou, China; ^10^Medical Genetics Centre, Guangdong Women and Children Hospital, Guangzhou, China; ^11^Pediatric Intensive Care Unit, Affiliated Bayi Children's Hospital General Hospital of the People's Liberation Army, Beijing, China

**Keywords:** Phelan-McDermid syndrome, 22q13 deletion, *SHANK3*, novel mutation, genetic

## Abstract

**Background:**

PhelanrMcDermid syndrome (PMS) is an uncommon autosomal dominant inherited developmental disorder. The main characteristics are hypotonia, intellectual disability, autism spectrum disorder, autism-like behaviors and tiny facial deformities. Most cases are caused by the deletion of the 22q13 genomic region, including the deletion of *SHANK3*.

**Methods:**

Genetic and phenotype evaluations of ten Chinese pediatric patients were performed. The clinical phenotypes and genetic testing results were collected statistically. We analyzed the deletion of the 22q13 genomic region and small mutations in *SHANK3* (GRCh37/hg19) and performed parental genotype verification to determine whether it was related to the parents or was a novel mutation.

**Results:**

The age of the patients diagnosed with PMS ranged from 0 to 12 years old. Nine of the pediatric patients experienced Intellectual Disability, language motion development delay and hypotonia as prominent clinical features. One subject had autism, two subjects had abnormal electroencephalogram discharge and one subject was aborted after fetal diagnosis. Three patients had a *SHANK3* mutation or deletion. All but the aborted fetuses had intellectual disability. Among the ten patients, a deletion in the 22q13 region occurred in seven patients, with the smallest being 60.6 kb and the largest being >5.5 Mb. Three patients had heterozygous mutations in the *SHANK3* gene.

**Conclusion:**

All ten patients had novel mutations, and three of these were missense or frameshift mutations. For the first time reported, it is predicted that the amino acid termination code may appear before protein synthesis. The novel mutations we discovered provide a reference for clinical research and the diagnosis of PMS.

## Introduction

PhelancMcDermid syndrome [PMS, Online Mendelian Inheritance in Man (OMIM) 606232], also known as 22q13.3 deletion syndrome, is an adjacent gene disorder caused by long arm deletion of chromosome 22. It is a rare developmental disorder with autosomal dominant inheritance (1). Chromosomal abnormalities include simple terminal deletions, interstitial deletions, ring chromosomes, duplications and translocations (2). A pathogenic nucleotide variant in this critical gene can also cause PMS (3). It is characterized by different clinical symptoms with varying degrees of severity, such as severe speech retardation, neonatal hypotension, Intellectual Disability or impairment, intellectual disability (ID), autism spectrum disorder (ASD), seizures, heart defects, recurrent upper respiratory tract infections, gastroesophageal reflux and metachromatic leukodystrophy (4–7). Until now, the true prevalence of PMS is unclear for us, according to the data from the Foundation of PMS, more than 2,000 people have been diagnosed with PMS worldwide. Among the children with PMS, the proportion of ASD children was 84% (8, 9).

The 22q13.3 deletion region involves multiple OMIM pathogenic genes, such as *ALG12, MLC1, TRMU, TUBGCP6, TYMP, CHKB, SBF1, ARSA*, and *SHANK3* (9). Particularly noteworthy genes include *CYB5R3* and *MPPED1* which was in 22q13.2; and *NUP50, KIAA1644, FBLN1, PARVB, C22orf9, WNT7B, TRMU, ATXN10*, microRNAs hsa-let7, hsa-let-7a-3, hsa-mir-1249 in 22q13.31 (2).

Mechanisms leading to the deletions, such as unbalanced translocations, simple deletions and circular chromosomes are highly variable (10). The disease is caused by a distal deletion of chromosome 22q13, which includes the SHANK3 gene in most cases. The reason for this syndrome is: the deletion and mutation of chromosome 22q13.3 lead to the loss of SHANK3 functional copy, and the deletion of the chromosome may be caused by one of the parents' balanced translocations or *de novo*. As we know, the pathogenic mutation of *SHANK3* is almost *de novo* (11, 12). Furthermore, in our study, the deletion of 22q13 appeared *de novo*, and the deleted genomic segment size ranged from hundreds of kilobases (kb) to over nine megabases (Mb).

*SHANK3* encodes glutamate postsynaptic density scaffold protein, then plays a key role in synaptic function by regulating dendrite formation. It is generally believed that SHANK3 gene is the main candidate gene for neurological characteristics of the syndrome, which is located in the 22q13 region. If SHANK3 leads to most neurological abnormalities in these patients, it means that the proximal deletion of SHANK3 may have a milder phenotype. However, this phenomenon is largely concealed by the end deletion of SHANK3 (3).

Due to the rarity of this syndrome, the reporting about pediatric patients, especially *de novo* cases, is still insufficient (13, 14). We collected data from ten pediatric patients with PMS from ten hospitals in China for statistical and genetic evaluation of clinical phenotypes, genetic analysis of 22q13 deletions and *SHANK3* minor mutations. We also carried out parental genotype verification to determine whether the mutations were related to their parents or novel.

## Materials and Methods

### Subjects

Ten pediatric patients from ten hospitals in China were selected for clinical and genetic analysis. The inclusion criteria were: (1) patients diagnosed with PhelanpMcDermid syndrome; (2) the patientomeosed with Phe incluscooperated with relevant examinations. Informed consent was obtained from the patients' families.

### Next-Generation Sequencing

The coding regions of related genes were amplified using the method based on target region capture and sequenced using the next-generation sequencing platform (ABI SOLiD sequencer). This detected and analyzed copy number (CN) variations of genes within the detection range (GRCh37/hg19) of the pediatric patients and their parents.

## Results

The study included four males (40%) and six females (60%), with ages ranging from 0 to 12 years old. Speech delay, motor delay, Intellectual Disability or impairment and ID were the main clinical symptoms (Table 1). Case 1: female, 9 years old, with low intelligence, poor motor balance, fine motor skills, poor language communication skills and unable to take care of herself. Case 2: female, 6 years old. She can call her parents when she is more than 2 years old. So far, she can only repeat words and barely understand adult instructions. She has depigmentation spots in her left ear, hairy back, unsupported autism screening, and low neuropsychological evaluation. A large number of abnormal EEG waves, ASD to be discharged. Case 3: male, 6 years old, with normal motor function, obviously backward language development and mild abnormal intelligence test. Case 4: male, 12 years old, with brain dysplasia, mental retardation and abnormal behavior. EEG shows abnormal discharge in frontal part. Case 5: female, 10 years old, growth retardation, mental retardation, dystonia, autism. Case 6: male, 7 years old, mentally retarded. Case 7: male, 1-year-old, with low muscle tone, backward intellectual development and motor development. Case 8: female, 5 years old, has been slow in learning and movement, unable to speak. Ultrasound indicates left polycystic kidney. Case 9: female, 2 years old, growth retardation, encephalomalacia (paraventricular), backward language development, unable to walk autonomously, insensitive hearing, brain MRI indicates paraventricular malacia, blood examination indicates that T3 and T4 are increased and TSH is decreased. Case 10: 0 years old (prenatal), routine three-dimensional ultrasound examination during maternal pregnancy showed fetal pericardial effusion and peritoneal effusion.

**Table 1:** Clinical phenotype in ten individuals with PMS.

**Patient**	**1**	**2**	**3**	**4**	**5**	**6**	**7**	**8**	**9**	**10**
Gender	Female	Female	Male	Male	Female	Male	Male	Female	Female	Female
Age (years)	9	6	6	12	10	7	1	5	2	0
Speech delay	+	+	+	+	+	+	+	+	+	–
motor delay	+	+	–	+	+	+	+	+	+	–
mental retardation	+	+	+	+	+	+	+	+	+	–
or impairment
ID	+	+	+	–	+	+	+	+	+	–
ASD	–	–	–	–	+	–	–	–	–	–
EEG	–	+	–	+	–	–	–	–	–	–
Dysmorphic facial	–	+	–	–	–	–	–	–		–
features
MRI	–	Brain MRI	–	–	–	–	–	–	Brain MRI	–
		normal							indicated	
									paraventricular
									leukomalacia
Others	–	–	–	–	Hypotonia	Hypotonia	Hypotonia	Polycystic	Blood	Fetal
								kidney on	indicates T3,	ultrasound
								the left	T4 high,	showed
								side	TSH low	pericardial
										effusion and
										abdominal
										effusion.

All the above 10 cases were found to have abnormal clinical manifestations, mainly neurological symptoms, without family history. Therefore, combined with the clinical symptoms and family history, the analysis was conducted to improve the genetic test to confirm the genetic relationship.

### Results of Genetic Testing

**Figure 1:**
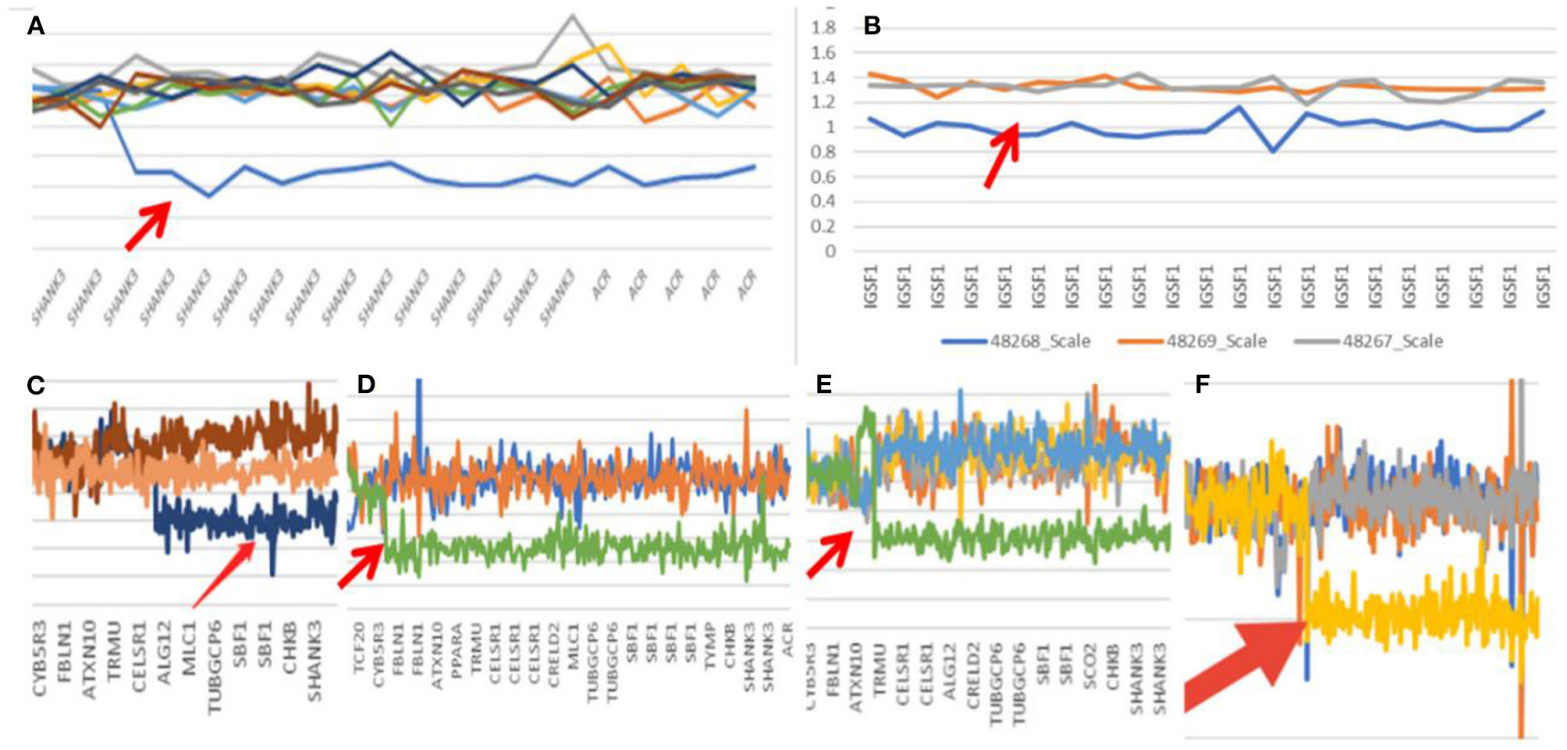
Gene mutation map (Arrow shows mutation; **A**: case one; **B**: case two; **C**: case three; **D**: case seven; **E**: case eight; **F**: case nine).

In Case 1, the 22q13.33 region had a deletion of at least 60.6 kb, the breakpoints of the deletion start at chr22:51123013 and end at chr22:51183635,included exons 9–23 of the *SHANK3* gene. This terminal deletion may occur on the paternal chromosome. Data and quantitative polymerase chain reaction verification experiments showed that the pediatric patient's parents did not carry this mutation (Figure 1A).

In Case 2, the 22q13.3 region of the paternal chromosome showed a heterozygous deletion with a size of at least 4.43 Mb. The breakpoints of the deletion start at chr22:46751341 and end at chr22:51183635. This terminal deletion may occur on the paternal chromosome. The pediatric patient's parents did not carry this CN mutation (Figure 1B).

In Case 3, the 22q13.3 region had a deletion of at least 886 kb. The breakpoints of the deletion start at chr22:50297486 and end at chr22:51183635. This terminal deletion may occur on the paternal chromosome. The sequencing data showed that neither of the pediatric patient's parents carried this mutation (Figure 1C).

In Case 4, the *SHANK3* gene heterozygous variant c.3727delG (p.A1243Pfs^*^57) was found. Simultaneous sequencing data showed that the pediatric patient's parents did not carry this mutation.

In Case 5, a deletion of at least 886 kb was found in the 22q13.3 region. The breakpoints of the deletion start at chr22:50297486 and end at chr22:51183635. This terminal deletion may occur on the paternal chromosome. The sequencing data showed that the pediatric patient's parents did not carry these mutations.

**Figure 2:**
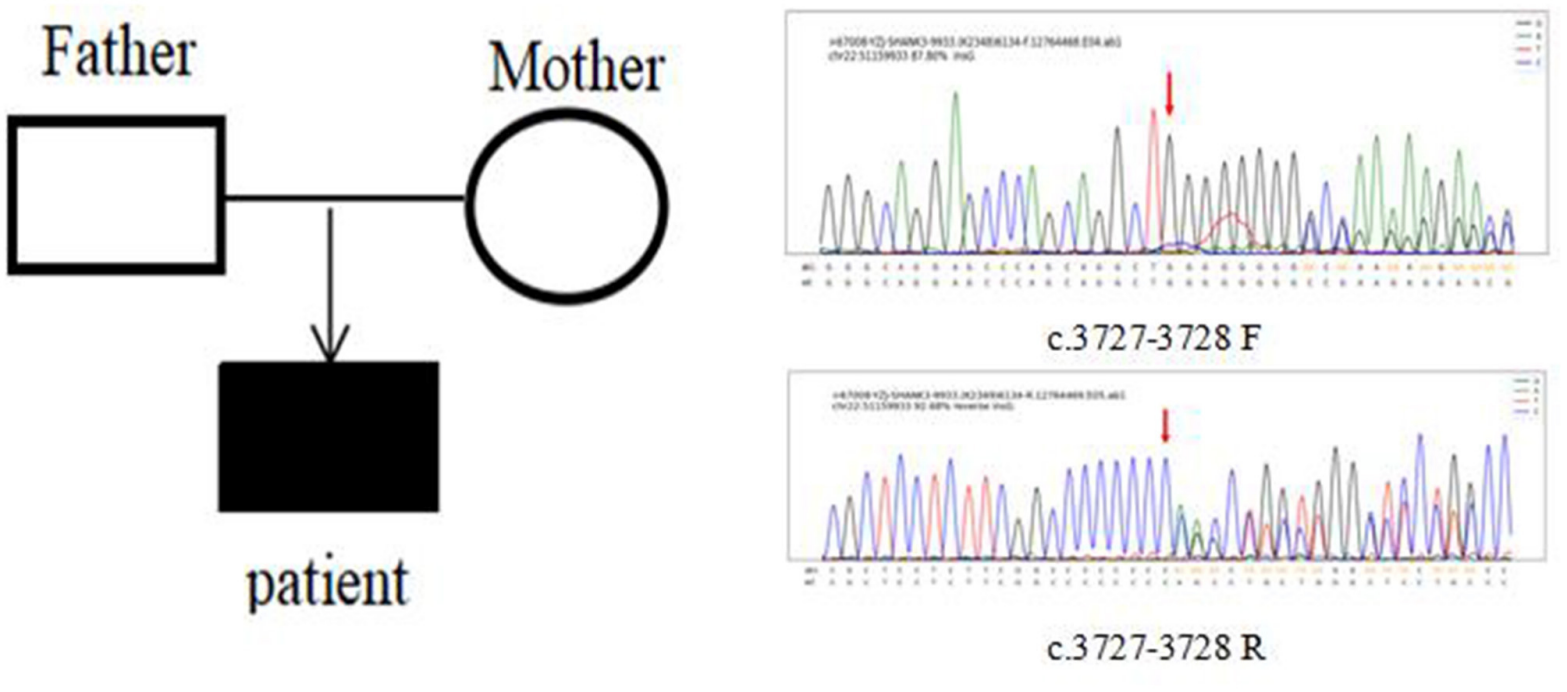
Case 6 children and parents with SHANK3 c.3727-3728 (patient heterozygous insertion F, R).

In Case 6, the *SHANK3* gene heterozygous variant c.3727dup (p.A1243Gfs^*^69) was found. The verification experiment showed that the pediatric patient's parents did not carry the mutation (Figure 2).

In Case 7, a deletion of at least 5.5 Mb in the 22q13.3 region was found. The breakpoints of the deletion start at chr22:45680895 and end at chr22:51183635. This terminal deletion may occur on the paternal chromosome. Sequencing data showed that neither of the pediatric patient's parents carried these mutations (Figure 1D).

In Case 8, there was a deletion of at least 4.45 Mb in the 22q13.3 region. The breakpoints of the deletion start at chr22:46731662 and end at chr22:51183635. This terminal deletion may occur on the paternal chromosome. The verification experiment showed that neither of the pediatric patients' parents carried this mutation (Figure 1E).

**Figure 3:**
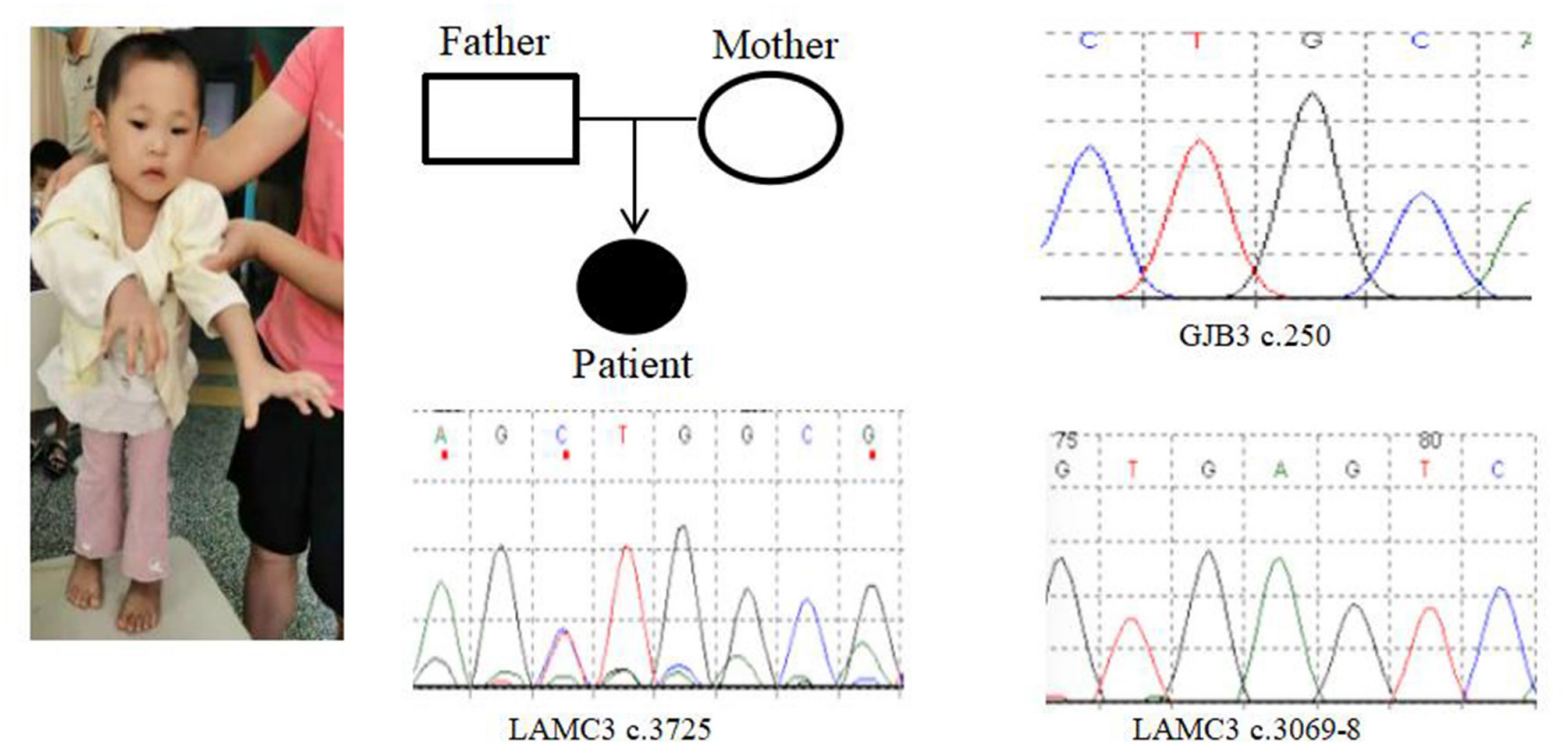
Case 9 GENE mutation map (patient with GJB3c.250heterozygous mutation; LAMC3c.3069-8_ heterozygous mutation; LAMC3c.3725heterozygous mutation).

In Case 9, there was a deletion of at least 5.3 Mb in the 22q13.3 region. The breakpoints of the deletion start at chr22:45898866 and end at chr22:51183635. This terminal deletion may occur on the paternal chromosome. The verification experiment showed that neither of the pediatric patient's parents carried this mutation (Figures 1F, 3).

In Case 10, the *SHANK3* gene heterozygous variant c.286G>A (p.V96I) was found. The verification experiment showed that the pediatric patient's parents did not carry the mutation (Tables 2, 3).

**Table 2:** Details of the 22q13.3 deletions in seven individuals with PMS.

**Patient**	**Ascertainment**	**Gender**	**Age**	**Variation**	**ACMGVariation**	**Array**	**Inheritance**	**Zygotic**	**Delation**
	**method**		**(years)**	**in chromosome**	**classification**	**coordinates**		**state**	**or duplication**
				**segment**		**(GRCh37/hg19)**			**size (kb)**
1	*WES*	Female	9	22q13.33	Type 1 pathogenic mutation	chr22:5112301 3−51183635	*De novo*	X1	60.6 KB
2	*WES*	Female	6	22q13.3	Type 1 pathogenic mutation	chr22:46751341- 51183635	*De novo*	X1	>4.43 Mb
3	*WES*	Male	6	22q13.3	Type 1 pathogenic mutation	chr22:50297486- 51183635	*De novo*	X1	>886 Kb
5	*WES*	Female	10	22q13.3	Type 1 pathogenic mutation	chr22:50297486-51183635	*De novo*	X1	>886 kb
7	*WES*	Male	1	22q13.3	Type 1 pathogenic mutation	chr22:45680895-51183635	*De novo*	X1	>5.5 Mb
8	*WES*	Female	5	22q13.3	Type 1 pathogenic mutation	chr22: 46731662−51183635	*De novo*	X1	> 4.45 Mb
9	*WES*	Female	2	22q13.3	Type 1 pathogenic mutation	chr22: 45898866-51183635	*De novo*	X1	>5.3 Mb

**Table 3:** Details of the *SHANKS* deletions in three individuals with PMS.

**Patient**	**Ascertainment**	**Gender**	**Age**	**Gene**	**ACMGVariation**	**GRCh37/hg19**	**Variant**	**Amino**	**Protein**	**Zygotic**	**Variant**
	**method**		**(years)**		**classification**	**location**	**type**	**acid change**	**change**	**state**	**type**
4	*WES*	Male	12	*SHANK3*	Type 2 possible pathogenic mutation	chr22: 51159933	*De novo*	c.3727delG	p.A1243Pfs*57	Heterozygous variants	Frameshift
6	*WES*	Male	7	*SHANK3*	Type 1 pathogenic mutation	chr22: 51159933	*De novo*	c.3727dup	p.A1243Gfs*69	Heterozygous variants	Frameshift
10	*WES*	Fetal	0	*SHANK3*	Type 3 unknown significance	chr22: 51115068	*De novo*	c.286G>A	p.V96I	Heterozygous variants	Missense

## Discussion

The analysis found all ten cases were *de novo*. The novel mutations we discovered provide a reference for clinical research and the diagnosis of PMS.

A previous study showed that a balanced translocation from one parent without no symptoms could lead to symptomatic imbalances translocation in children (15).

The 22q13 deletion can be detected in chorionic villi, percutaneous umbilical blood samples and amniotic fluid. In postnatal studies, it is usually difficult to find the absence due to its subtlety. If the banded chromosome test was suspect the 22q13 deletion, fluorescence *in situ* hybridization using a probe specific for 22q13 or comparative genomic hybridization microarray analysis should be performed to confirm this finding (16, 17). We reported a case of prenatal fetal ultrasound that showed pericardial effusion and ascites, and genetic testing suggested PMS, resulting in the fetus being aborted.

PMS is very subtle at birth and should be considered in all cases of unexplained neonatal hypotonia (16). We have proposed that prenatal diagnosis is essential for normal parents who have the affected children, the reason was the theoretical possibility of parental germline mosaicism.

The size of the deleted segment was highly variable in this study, ranging from 45.8 kb to 9.1 Mb. Those deletion sizes had been associated with the major neurological symptoms of PMS, larger deletions were associated with increased likelihood of dysmorphic features and medical comorbidities, while small deletions or SHANK3 pathogenic variants correlated with autism spectrum disorder, seizures, hypotonia, sleep disturbances, abnormal brain MRI, gastroesophageal reflux, and certain dysmorphic features (7). Sarasua et al. confirmed the trend correlating larger deletions with more severe clinical presentations and smaller deletions with autism spectrum disorder (2), but it cannot completely explain the clinical variability seen among individuals. Droogmans et al. did not find a relation between the deletion size and the severity of the phenotype (4), we also found that the clinical phenotype did not clearly show a close relationship with the size of the deletion. In our study, all of the cases (except case ten) had language and mental retardation, but only one case was clearly diagnosed with ASD, we considered it be associated with our relatively small samples.

The study found that skill loss occurs most frequently in the middle of childhood in PMS patients, mainly affecting motor and self-help skills. In addition, 43% of PMS patients have experienced recession, which is of potential significance to those who take care of them. Providers may consider screening or monitoring skills that may be lost in the middle term of children. Additionally, 43% of PMS patients have experienced regression, which is of potential importance for their caregivers, it may consider the possibility of screening or monitoring for possible loss of skills in middle childhood. PMS can be determined from clinical determination of the loss of skill which will prompt referral for genetic testing. The intervention measures may help maintain skills in later childhood or restore skills after degradation, and the importantly thing is how to develop and apply those interventions (8).

In order to reduce frustration and accelerate language development, we can early apply expanded (such as computer-assisted) pattern of communication, and promote the oral language using (18). A good equilibrium between the development or adaptation and limitations of the PMS patients, and environmental demands is indispensable to ensure the individualutewellbeing (19).

## Conclusion

PMS is often detected and diagnosed at the pediatric stage. The phenotype of these traits varies by age in assessment and deletion size, but is generally not determined by the parent of origin of the affected chromosome. Gene deletions in PMS patients have no obvious common breakpoints and tend to vary widely, and structural variants include terminal and mesenchymal deletions and duplications at 22q13 (2). These ten cases are the first reported new mutations, providing a reference for clinical research and treatment. Three of the mutations are frameshift or missense mutations, which are predicted to cause the amino acid termination code to appear before protein synthesis. This research provides a method for a more comprehensive clinical diagnosis of PMS patients using genetic analysis.

## Data Availability Statement

The original contributions presented in the study are included in the article/supplementary material, further inquiries can be directed to the corresponding author/s.

## Ethics Statement

The studies involving human participants were reviewed and approved by Guangdong Provincial People's Hospital. Written informed consent to participate in this study was provided by the participants' legal guardian/next of kin. Written informed consent was obtained from the minor(s)' legal guardian/next of kin for the publication of any potentially identifiable images or data included in this article.

## Author Contributions

LC, Z-yY, S-rH, and Y-mL designed the study, wrote the manuscript, and assessed the individual's pictures of the morphological analysis. XW, X-yW, D-zC, X-kY, J-bZ, ZR, HL, ZP, H-kD, and Z-cF analyzed and interpreted the genetic and clinical data. All authors read and approved the final manuscript.

## Funding

This work was supported by the National Natural Foundation of China (project number: 81671529), the Natural Science Foundation of Guangdong Province (project number: 2020A1515010511), and the National key R&D Program of China (2018YFC1002600).

## Conflict of Interest

The authors declare that the research was conducted in the absence of any commercial or financial relationships that could be construed as a potential conflict of interest.

## Publisher's Note

All claims expressed in this article are solely those of the authors and do not necessarily represent those of their affiliated organizations, or those of the publisher, the editors and the reviewers. Any product that may be evaluated in this article, or claim that may be made by its manufacturer, is not guaranteed or endorsed by the publisher.
